# Supplementary material for: Why the long face? Comparative shape analysis of miniature, pony, and other horse skulls reveals changes in ontogenetic growth
Source: PeerJ. 2019 Sep 16;7:e7678. doi: 10.7717/peerj.7678 (PMC6752190; doi:10.7717/peerj.7678)
Supplement: Supplemental Information 1 — This document contains: Table S2: Description of the landmarks, including position, type, and to which module it belongs, collected on each cranium. Table S3: Average wither height in cm for each breed, with reference. Figure S1: Depiction of landmarks used in this study. Figure S2: Principal component analysis of ontogenetic series of Shetland pony and Welsh specimens. Figure S3. Ontogenetic skull shape changes from juvenile to adult stage in thin-plate-spline representation. [file peerj-07-7678-s007.docx]

SUPPLEMENTARY MATERIAL

**Comparative shape analysis of pony and regular sized horse skulls**

Laura Heck^1^, Marcelo R. Sánchez-Villagra^1^, Madlen Stange^2^

^1^ Palaeontologisches Institut und Museum, Universität Zürich, Karl-Schmid-Strasse 4, 8006 Zürich, Switzerland

^2^ Redpath Museum, McGill University, 859 Sherbooke Street West, Montreal, QC, H3A 2K6, Canada

Corresponding Author:

Madlen Stange^2^

859 Sherbooke Street West, Montreal, QC, H3A 2K6, Canada

Email address: stange.madlen@gmail.com

# Contents

Table S2: Description of the landmarks, including position, type, and to which module it belongs, collected on each cranium.

Table S3: Average wither height in cm for each breed, with reference

Figure S1: Depiction of landmarks used in this study.

Figure S2: Principal component analysis of ontogenetic series of Shetland pony and Welsh specimens.

Figure S3. Ontogenetic skull shape changes from juvenile to adult stage in thin-plate-spline representation.

**Table S1:** The raw data table is provided as an Excel sheet due to its large size. The Table contains information where the skulls are deposited, museum identifiers, classification codes, ratios for paedomorphic feature assessment and the three-dimensional shape coordinates.

**Table S2:** Description of the landmarks, including position, and type collected on each cranium.

| **Cranium** | **Position** | **Type** |
| --- | --- | --- |
| **1 - 2** | Posterior tip of the upper third incisor | II |
| **3 - 4** | Posterior most point of the nasal-premaxilla suture | I |
| **5 - 6** | Premaxillary-maxillary-nasal suture | I |
| **7 - 8** | Dorsoposterior tip of the infraorbital foramen | II |
| **9 - 10** | Anterior tip of the facial crest | II |
| **11** | Nasion, nasal-frontal suture, midline | I |
| **12 -13** | Junction of the lacrimal, maxilla, and nasal sutures | I |
| **14 - 15** | Zygo-lacrimal suture on the orbital margin | I |
| **16 - 17** | Lacrimal-frontal suture on the orbital margin | I |
| **18 -19** | Supraorbital foramen | II |
| **20 - 21** | Anterior tip of the zygo-temporal suture | I |
| **22 - 23** | Posterior tip of the zygo-temporal suture | I |
| **24 - 25** | Dorsal tip of the frontal-temporal suture | I |
| **26 - 27** | Ventro-posterior tip of the zygomatic process | II |
| **28 - 29** | Dorsal most point of the vertically orientated posterior margin of the zygomatic process | II |
| **30 - 31** | Ventro-lateral most point of squamous part of temporal bone | II |
| **32** | Anterior tip of the occipital triangle | I |
| **33** | Posterior tip of the nuchal crest | II |
| **34 - 35** | Dorsolateral tip of the nuchal crest | II |
| **36** | Dorsal most point on the margin of the foramen magnum | II |
| **37** | Point between first incisors from ventral side | II |
| **38 - 39** | Posterior most tip of the premaxillary-maxillary suture, ventral | I |
| **40 - 41** | Anterior tip of the second premolar | II |
| **42** | Posterior most point of the incisive canal | II |
| **43** | Posterior tip of the palatine process of the incisive bone | I |
| **44** | Posterior tip of the palatine-palatine suture | I |
| **45 - 46** | Distal tip of the pterygoid hamulus | II |
| **47 - 48** | Anterior tip of the caudal alar foramen | II |
| **49** | Posterior tip of the vomer on the midline | II |
| **50 - 51** | Medial tip of the mandibular fossa | II |
| **52 - 53** | Canal for hypoglossal nerve | II |
| **54 - 55** | Fossa medial of the paracondylar process | II |
| **56 - 57** | Distal tip of the paracondylar process | II |
| **58** | Ventral tip of the foramen magnum | II |
| **59 - 60** | Posterior most tip of the occipital condyle | II |

Type I: discrete juxtapositions of tissue types and Type II: maxima of curvature or other local morphogenetic processes [1].

**Table S3:** Average wither height in cm for each breed, with reference.

| **Breed** | **Average (cm)** | **Pony or regular sized breed** | **Reference** |
| --- | --- | --- | --- |
| **Anglo Norman** | 160 | regular | [2] |
| **Arab** | 148 | regular | [3] |
| **Belgian Draft** | 168 | regular | [4] |
| **Bosnian Pony** | 137 | Pony | [5] |
| **Clydesdale** | 175 | regular | [6] |
| **Exmoor Pony** | 123 | Pony | [7] |
| **Falabella** | 78 | Pony (Miniature) | [8] |
| **Grison** | 155 | regular | [9] |
| **German Riding Pony** | 148 | Pony | [10] |
| **Hannoverian** | 165 | regular | [11] |
| **Hackney** | 145 | regular | [12] |
| **Holstein** | 170 | regular | [13] |
| **Hungarian** | 165 | regular | [14] |
| **Huzule** | 139 | Pony | [15] |
| **Icelandic Horse** | 140 | Pony | [16] |
| **Kladrubian** | 167 | regular | [17] |
| **Konik** | 135 | Pony | [18] |
| **Lipizzan** | 155 | regular | [19] |
| **Mongolian** | 132 | Pony | [20] |
| **Norik/ Pinzgauer** | 157 | regular | [21] |
| **Nonius** | 160 | regular | [22] |
| **Oldenburgian** | 165 | regular | [23] |
| **Scottish pony** | 140 | Pony | [24] |
| **Shetland Pony** | 97 | Pony (Miniature) | [25] |
| **Shire** | 178 | regular | [26] |
| **Suffolk** | 166 | regular | [27] |
| **English Thoroughbred** | 163 | regular | [28] |
| **Trakehner** | 165 | regular | [29] |
| **Welsh** | 130 | Pony | [30] |

Please note that the terminological transition between ponies and regular breeds is not strictly determined by size but also by temperament (lively, child-friendly) and phenotype of the head: pony-like – “friendly-looking, pleasant manner”, and horse-like – “good stamina” are common attributes to discriminate between the two.

**
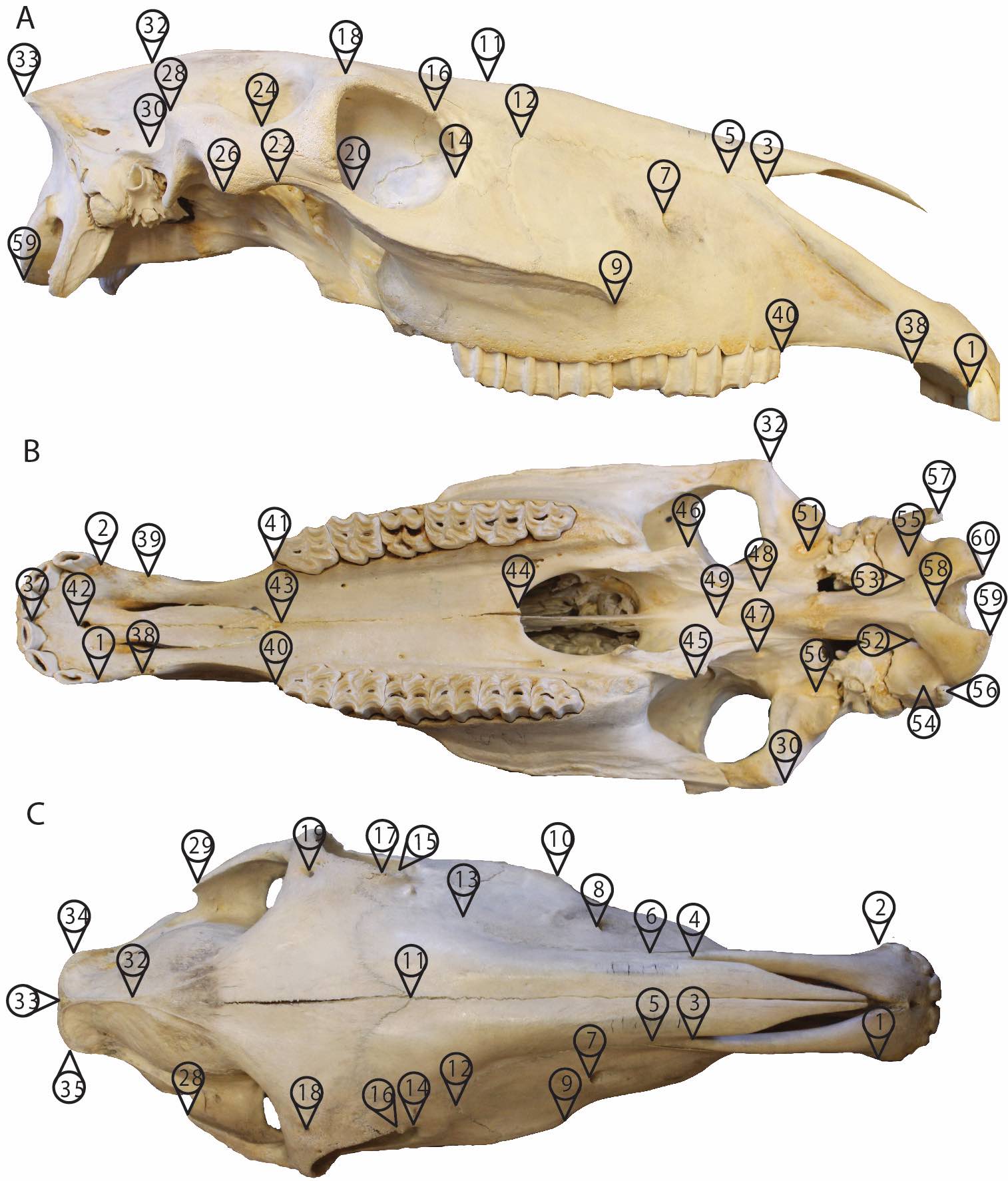
**

**Figure S1:** A total of 60 landmarks used in this study, first used in [31] shown on the A) lateral B) ventral C) dorsal side of the cranium of a normal sized horse breed (for a detailed description of the landmarks see Table S2).

**
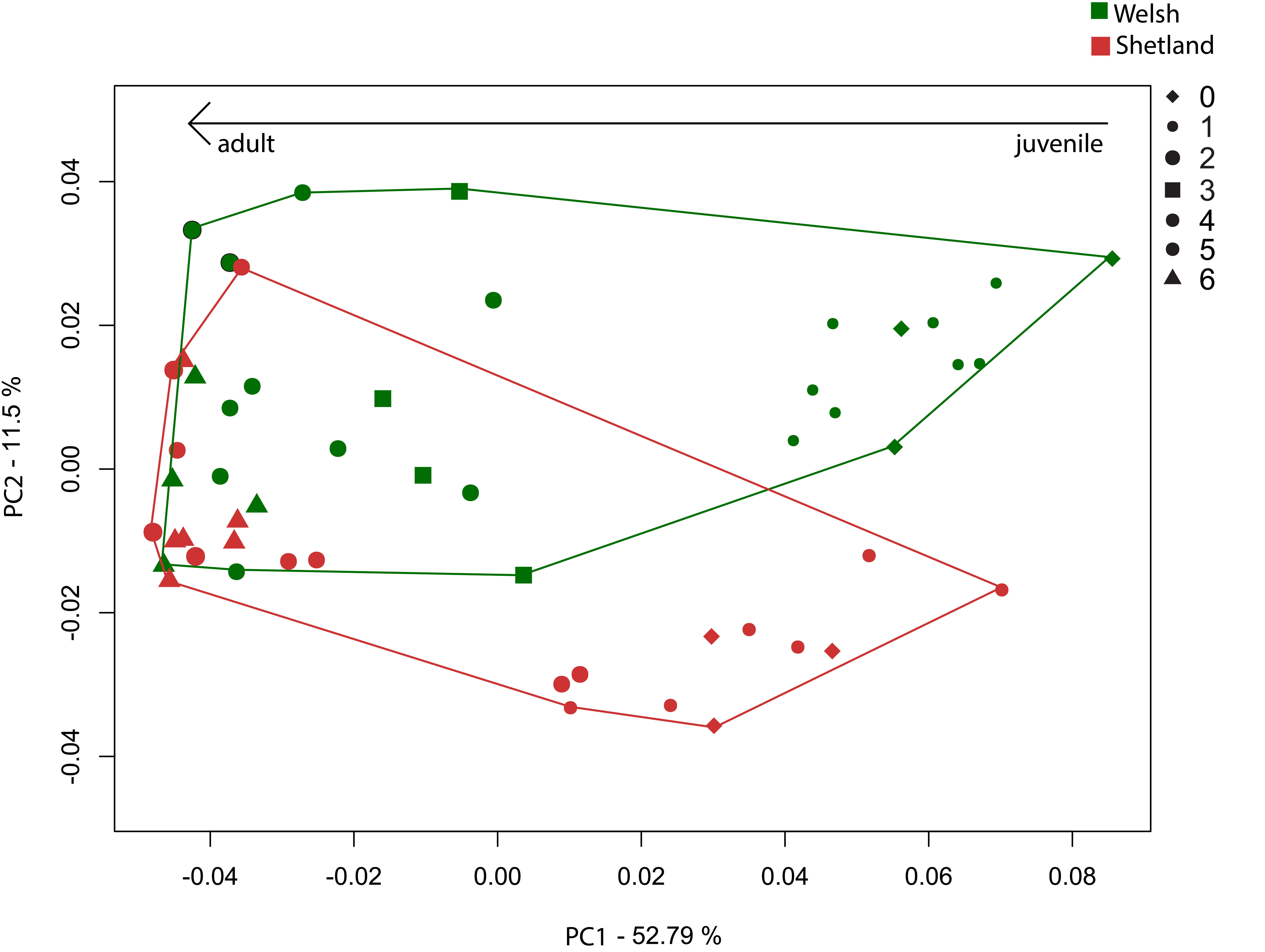
**

**Figure S2:** Principal component analysis of ontogenetic series of one pony – Welsh (green) – and one miniature breed – Shetland (red).

#

**Figure S3:** Ontogenetic skull shape changes from juvenile to adult stage in thin-plate-spline representation.

# References

1. Bookstein FL. Introduction to methods for landmark data. In: Rohlf FJ, Bookstein FL, editors. Prooceedings Michigan Morphometrics workshop, vol. Volume 2. Ann Arbor: The University of Michigan Museum of Zoology; 1990. p. 216–225.

2. Stud-Book Selle Français. Studbook Anglo Norman; 2017.

3. Arabian Horse Association. 2017. Studbook Arab.

4. Trekpaard. 2017. Studbook Belgian Draft..

5. Gesellschaft der Freunde FuZdBP. 2017. Studbook Bosnian Pony.

6. Clydesdale Horse Society. 2017. Studbook Clydesdale.

7. The Exmoor Pony Society. 2017. Studbook Exmoor Pony.

8. The Falabella Miniature Horse Association. 2017. Studbook Falabella.

9. Pferdezucht-Genossenschaft Graubünden. 2017. Studbook Grisons.

10. Deutsche Reiterliche Vereinigung (FN). 2017. Studbook German Riding Pony.

11. Hannoveraner Verband. 2017. Studbook Hannoverian.

12. American Hackney Horse Society. 2017. Studbook Hackney.

13. Verband der Züchter des Holsteiner Pferdes e.V. 2017. Studbook Holstein.

14. Bene S, Kecskes B, Polgar P, SZABo F. Comparison of live weight and body measurements of adult brood mares from different genotypes in Hungary. J. Cent. Eur. Agric. 2014;15:1–11.

15. Polish Horse Breeders Association. 2017a. Studbook Huzule.

16. International Federation of Icelandic Horse Associations. 2017. Studbook Icelandic Horse.

17. Stud Kladruby nad Labem. 2017. Studbook Kladrubian.

18. Polish Horse Breeders Association. 2017b. Studbook Konik.

19. Zuchtorganisation Gestüt Lipica. 2017. Studbook Lipizzan

20. Wikipedia Contributors. Mongolian horse [Internet]. Wikipedia, The Free Encyclopedia; Available from: https://en.wikipedia.org/w/index.php?title=Mongolian_horse&oldid=841436531

21. Druml T, Baumung R, Sölkner J. Morphological analysis and effect of selection for conformation in the Noriker draught horse population. Livest. Sci. 2008;115:118–28.

22. Nonius Lótenyésztő Országos Egyesület. 2017. Studbook Nonius.

23. Oldenburger Verband. 2017. Studbook Oldenburgian.

24. IG Highland Pony "Der Clan" e.V. 2017. Studbook Scottish Pony.

25. Schweizerischer Shetlandponyverband. 2017. Studbook Shetland Pony.

26. Shire Horse Society. 2017. Studbook Shire.

27. Suffolk Horse Society. 2017. Studbook Suffolk.

28. The Jockey Club. 2017. Studbook English Thoroughbred.

29. Trakehner Verband. 2017. Studbook Trakehner.

30. The Welsh and Cob Society. 2017. Studbook Welsh.

31. Heck L, Wilson LAB, Evin A, Stange M, Sánchez-Villagra MR. 2018. Shape variation and modularity of skull and teeth in domesticated horses and wild equids. Frontiers in Zoology 15. DOI: 10.1186/s12983-018-0258-9.
